# New Fields of Medicine

**Published:** 1916-07

**Authors:** T. D. Crothers

**Affiliations:** Hartford, Conn.


### New Fields of Medicine.

BY DR. T. D. CROTHERS, HARTFORD, CONN.

Everywhere it is apparent that there is a breaking up of old methods and systems of practice. The physician sits in his office, and is only called on when the patient has reached alarming states of pronounced diseases. An early period when the physician could have been of real service has passed by. During this time the patient has treated himself with proprietary drugs, household remedies and perhaps called oñ some quack doctor who has alarmed him, if nothing else. Then in despair, at the failure of all these efforts, the physician is called in.

The diagnosis which should have been clear before is greatly complicated by the effects of drugs and other conditions of depression and fear and the actual condition does not appear for several days. Then some acute inflammatory conditions appear, or some bacterial poisons coupled with a variety of complex symptoms both functional and organic, that should not have existed if the case had been treated properly at the beginning. Days and weeks go on, and the patient finally recovers. The physician is conscious had he been called in the early stages, the results would have been different.

On the other hand, a modern family who realizes that a physician's best counsel and advice are along the line of prevention, find a doctor in whom they have confidence, and engage him to look after their health interests. He calls often at the house and welcomes their visits to his office, where the subject of the family health is discussed in detail.

There is no question here of short cuts to health, no specific or proprietary drugs are put into service and no irregular who claims marvelous capacity is consulted. The family are surprised and delighted at their freedom from disease and ill health, compared with their neighbors who have no family physician employed in this way.

The physician takes pride in his judicial position as counsellor and rarely has to conduct a case of fever or disease that require his attention daily. The physician himself is a model of good health, good judgment and excellent common sense. He is not running after poverty cases or conducting a dispensary or boasting of his hospital connections. He attends to business daily. When a pauper case appears, he treats him kindly and suggests means and ways for relief that will not burden him or the neighborhood. The physician is a church man, appearing regularly at the weekly services, and showing, by his example, interest and sympathy for all efforts that shall help men and women.

He is a member of the lodges where the more social interests are sought and promoted. While he may not take an active part, he is conservative, sympathetic and helpful. Such a man soon enlists a confidence of the best people in the community, who often come to him for counsel on matters semi-scientific and social, and in this way he reaches out to a clientage beyond that of the clergyman or lawyer, a clientage that pays in a great many ways that are not measured by dollars and cents.

There are a number of physicians who are doing this and who are every year rising above the old-time practice of running about from place to place and from house to house, dealing out medicine and boasting of their success. Men of the old type never take an active part in reform work to stamp out evils, that involve controversy and discussion. Such men are too busy to attend church. The lodges do not attract them. They can't afford to oppose the open saloon. In politics they are conservative, first on one side and then on the other, and are never known exactly as to their political convictions. They are veritable Mr. Face-Both-Ways. The only personality they exhibit is a critical spirit of others in the same profession, and a positiveness about the results of their work which others do not share.

They go down through the years, following the old line; their influence and work are always more or less uncertain in the public mind. A few people are very warmly attached to them, but the great majority are uncertain and not in touch with them.

Now, this is not the modern field of medicine that is coming. It is not meeting the requirements of a higher intelligence and slowly awakening consciousness that the doctor's work is something more than mere druggist counter business.

The coming physician is already here. The numbers are few, and they are so widely scattered that they have not yet been recognized. The great rank and file of the profession have been running down, deteriorating for the last quarter of a century. The new men taking the place of the old have only gone forward a little in surgery and diagnosis; but beyond this, about the same thing exists, and with it a constant decrease in income.

It is now estimated that the average income of the 130,000 physicians in the United States is less than $1000 per year. A

large number do not make half this amount, and the cry has gone up that the profession is overcrowded, that the medical colleges are turning out very poor men. The latter statement is partially true. The former is not so. The demand for physicians today is greater than ever, but the persons to meet this demand are not in sight.

Armies of medical men are struggling far back in the rear, while clergymen, reformers, women and health officers are trying to institute new plans and methods of improving the health of the community, clearing up the centers of disease and the plague spots, teaching a better life and a return to the great principles of health and vigor. There is hardly a community in the country in which the really wide-awake reformers, not men who are pushing fads and new impractical ideas, but men who are urging better living, better thinking, better training, are physicians. They of all others should lead in work for the betterment of the race. They should be the real priests to direct the hygienic and health interests of the community.

Look over the health boards in towns and cities and you will observe that they are largely composed of laymen; of six or seven on the board, only one or two are physicians. The questions of health, which should be decided by them, are settled by others. Health inspectors and advisers of school matters, water boards, and many other things that concern the health interests of the community are in the hands of laymen.

In one instance, a health board composed of a saloon keeper as president, a grocer, a lawyer and a politician, the latter being in active charge, neglected to take care of the water supply of a town. A group of Italian laborers were permitted to camp on the watershed. As a result a typhoid fever epidemic broke out and two hundred cases with a mortality of over forty followed. If this health board had been composed of physicians familiar with their duties, no such a thing would have happened. In that town and in many others the physician is not considered to be anything more than a drug-dispenser or a plumber-surgeon. If any great questions of public interest come up, clergymen, lawyers and politicians are called in counsel.

The physician who, of all others, should be best trained to advise on great sanitary questions is ignored and considered no better than an average man. This is a great blunder. Physicians should be trained to be sanitarians as well as surgeons. They should be trained to recognize the great evils of the community and the practical physical remedies required for their removal.

Medical colleges go on the same old way, and the half-trained graduates have to learn by hard experience the great questions which the community is confronted with, and the remedial means most practical for their removal. In the eastern part of the country, where tradition and prestige hold absolute sway, it is difficult to break up old customs and habits, but in the South and West physicians have less obstacles to overcome. They could institute quickly new methods of treatment, new plans for counsel and consultation, and take prominence in every community as sanitarians in the highest sense of that word.

Organizations concerning the welfare of the people that do not include physicians as leaders will fail. That is the province, that is the work, that is the new field in which their studies and practice should give them greatest power.

In a Southwestern city, a sanitary inspector with a salary of $3000 a year was a former barkeeper. It is simply absurd to suppose that this former business could fit him for this most responsible one. There are no doubt many capable physicians who are practically starving in that city who could fill that position with credit to themselves and the community.

In another town the president of the health board is a mere figurehead, a retired physician who has made property in the rise of real estate. His duties are regulated by the clerks and assistants. An active, capable physician in that position, drawing a good salary, would show the results of his work in diminished sickness and mortality. These changes can be made in the South and West, and they constitute the real practice of the future.

The medical men of every county and State should insist that they be recognized in questions of health, vital statistics and other topics pertaining to the future welfare of the community. When these questions are understood and the physician comprehends the conditions, and insists on them being met, the community will very gladly follow, although it may be an effort in some places.

The psycho-therapeutic field, meaning advice and counsel to persons who are not sick in the ordinary meaning of that word, but who are troubled, distracted by events both social and financial, need help, medical advice and are anxiously waiting for someone. Today they call on clergymen and lawyers, receive religious counsel and legal advice as to what they can do, then go home dissatisfied, and worse than ever.

There is in every community persons of this class who need wise medical counsel and who would gladly pay for it if it could be had. There are physicians who could give it, but they must become students of this higher field of medicine. They must become leaders and not followers. A few of them are already in the field. This is another region white with the harvest, awaiting laborers.

There are other fields that call for medical counsel where physicians are sorely needed. Thus educational centers, the school systems in towns and cities, are beginning to recognize that the value of their work depends on a recognition of the physical laws of growth and development; that the physician should determine this, and not the teacher; that educational matters are physical as well as mental, and every school board should have physicians to look after the children and direct the methods of training, the surroundings and other topics that come along with the subject.

The great Eastern colleges with their athletic departments and medical men in charge give some intimations as to what degree medical training can be made useful in preparing medical men for the future. There is not a school in the country in which the medical man's services could not be used most practically, and yet they are rarely seen in educational circles.

Modern science shows that a great many of the diseases and defects common to every day life come from faulty nutrition, ignorance in the selection and preparation of foods. The diet kitchen of the humblest home and the largest hotels and restaurants are without system, direction and scientific guide. The work is done haphazard according to tradition and with little or no reference to the great laws of nutrition and diet.

Cooks are untrained, only in boiling, frying and combining foods according to flavors and morbid tastes. There is little or no progress in this, and little or no science. It is pure guess work and the following of blind rules. What a field there is here for physicians who should determine the diet requisite for school children, for workmen, for brain workers, for women and children; and yet they are seldom consulted.

When the public understand that a physician's knowledge on these topics is of practical economic value they will be only too ready to pay for it. When a physician understands that this field of chemistry is unoccupied and calling for doctors who can advise properly and clearly, he will prepare and occupy it.

In the commercial world, when the employer of large numbers of men realizes that the advice and counsel of the doctor regarding the employes, their conditions of health, and how they can be made more effectual and turn out larger products with less expense, the medical man will be essential to the successful prosecution of the business. Already this has begun. In several large industrial plants in this country, one or two physicians are constantly employed to look after the health of the men, to prevent accidents and treat injuries and determine questions of how the workmen can do the best work with the greatest uniformity and steadiness, and thus contribute to the prosperity of the company.

One of the great leaders at a manufacturers' convention said: "We employ two physicians, and their services have added immensely to the product and the success of our work. This I began as an experiment, and I have found it most profitable, and believe that no man employing over 100 men in any mechanical work can afford to be without the services of a physician."

This is another field that is white with the harvest, and the starving recent graduates do not realize or prepare to give services in this direction.

One of the great cotton mills of the South employs two physicians regularly to attend their operators and keep them in the best of health.

There is another field that physicians do not occupy to the extent of their ability; that is the question of pauperism, which confronts almost every large community in the country. What to do with the indigent, defective and degenerate classes is discussed by clergymen, women and reformers, and the physician is seldom ever called except to administer to the coarsest symptoms, as a druggist or surgeon.

In the almshouses all over the country no medical knowledge is called for in the housing and caring for them. It is a matter of economy. The cheapest help, the cheapest men are given positions to care for them. Here is a neglected field in which medical knowledge is pre-eminent.

Where these poor indigents are taken out into the country and treated from an intelligent, medical standpoint, they do not become the burden that the taxpayers complain of. There is a large army, which possibly may be increasing, of defectives, who if properly housed and medically directed could be made, to a large degree, self-supporting. What they need is scientific care, scientific direction and control, not founded on stupid theories or blundering selfishness. Trained physicians here would easily recognize the possibilities and capacities of these unfortunates, and know how far they can be utilized in supporting themselves, and contributing in some way to help others.

The profession has recognized that the epileptic, the insane need medical help, and have conceded that they can do more in this field than the laymen, but beyond that the same stupid blundering prevails and medical men very seldom have anything to do with it.

The great conclusion is that a proper medical training, meaning a knowledge of the physiology and anatomy of the human body and a general knowledge of the diseases and the laws which control their growth and decline, is a most essential knowledge to fit men to take care of others—to become helpful in the great struggle upward and outward.

The medical colleges have not caught sight of these new fields. They concentrate their efforts on bacteriological diseases, on surgical technique, on repairs and diagnosis and a great many other side issues, which while valuable in many ways, are not practically available in the realities of every-day life.

The treatment of the mind is rarely ever touched. Psychical therapeutics are unknown even in the great universities of this country, and a great many other topics, which the quacks with foresight and cunning are promoting, are ignored.

In reality the physician, instead of starving the first few years of his life, then struggling on and dying with only a small life insurance to his credit, should be the best paid man in the community. His knowledge is superior and special and the public need it, and some time not far away they will call for him to occupy fields unknown at present.

